# Design and Implementation of an Einsteinian Energy Learning Module

**DOI:** 10.1007/s10763-022-10348-5

**Published:** 2023-03-01

**Authors:** Shachar Boublil, David Blair, David F. Treagust

**Affiliations:** 1 School of Physics, University of Western Australia, Crawley, Perth, Australia; 2 School of Education, Curtin University, Perth, Australia

**Keywords:** Energy, Einsteinian physics, Photons, Energy-Mass equivalence, Student conceptions

## Abstract

The most famous equation in physics, *E* = *mc*^2^, is rarely introduced in middle school physics curricula. Recent research has shown that teaching Einsteinian concepts at the middle school level is feasible and beneficial. This paper analyses an Einsteinian energy teaching module for Year 8 students (13–14 years old), which encompasses the two fundamental energy formulas in modern physics, *E* = *mc*^2^ and *E* = *hf*. In the context of activity-based learning, the Einsteinian energy module relates to all the forms of energy in traditional school curricula. This study uses a design-based research approach within the Model of Educational Reconstruction framework. Modern experiments, historical events, and educational research helped us identify relevant Einsteinian energy concepts, activities, and assessments. The study included 22 students who participated in nine in-class Einsteinian energy lessons. Analysing results in the post-test showed a 31% mean increase from the pre-test, a clear and significant positive change in students’ conceptual understanding. The results demonstrated students’ ability to deal with very large and small constants of proportionality and physical concepts involved in the module.

## Introduction

Albert Einstein’s glorious year of 1905 contained two papers that changed how we understand energy. The first paper, based on Planck’s formula *E* = *hf*, determined that the nature of light is quantised as packets of energy which we now call photons. In the second article, “Does the inertia of a body depend upon its energy-content?” Einstein used his theory of relativity to determine that energy has mass. This idea differed from the accepted view at the time that, in physical reactions, mass is conserved (Einstein & Infeld, 1938). Two years later, in 1907, Einstein published a longer article proposing the formula *E* = *mc*^2^ that would change our conception of energy and inertia (Hecht, 2011).

We rarely teach Einsteinian physics at the middle school level (Pitts et al., 2014), and *E* = *mc*^2^, the most famous equation of the twentieth century, is not part of the middle school curriculum. The same is true for *E* = *hf*, a formula that relates the energy of a photon to its frequency. Einsteinian physics relies on knowledge beyond our direct human experience and requires expensive and precise experiments to prove its validity, partly explaining why teachers and curriculum authorities are unaware of how to introduce it (Kersting, 2019). Still, implementing Einsteinian physics curriculums is challenging for researchers and teachers (Treagust, 2021). This paper addresses some of the challenges of implementing an Einsteinian physics curriculum focused on the topic of energy.

There is an increased awareness of the need to develop modern science educational resources. Einsteinian physics topics such as nanotechnology, quantum physics and relativity are starting to be part of science curricula (Antti, 2010; Ayene et al., 2019; Boublil & Blair, 2023; Hoehn et al., 2014; Kersting & Blair, 2021; Milner-Bolotin & Johnson, 2017; Pitts et al., 2014). This study builds on previous studies by our research team on teaching Einsteinian physics concepts in middle school (Chouldhary et al., 2020; Kaur et al., 2017b, c; Kersting & Blair, 2021; Pitts et al., 2014). In this paper, we focus on Einsteinian energy, a topic that has not been widely addressed in the literature (Kneubil, 2020).

We examine the integration of the Einsteinian energy module into a selected school’s Year 8 science curriculum. Our module is based on the two equations, *E* = *hf* and *E* = *mc*^2^, and focuses on the physical concepts involved and conceptual understanding of equations which requires students to deal with the very large and small physical constants. Einsteinian energy may appear highly conceptual; however, we have designed an activity-based curriculum where concepts and mathematics are complemented with hands-on experiments. The embodied activities promote students’ physical intuition, visualisation techniques and analogy-making, which are vital to understanding modern experiments.

The following sections outline the reasoning for including Einsteinian energy in the curriculum. We first set forth the research framework and aims and research questions of our study. We follow this section with the research framework, which uses the *Model of Educational Reconstruction* (Duit et al., 2012) and *design-based research* (Canu & Duque, 2014; McKenney & Reeves, 2013a). This section also includes the data collection methods. We then highlight key Einstein-Energy concepts and activities required to construct a module for Year 8 students. The results section describes 22 students’ understanding of Einsteinian energy after the nine lessons centred on the two main equations. In the discussion section, we outline research-based design principles for learning.

### Einsteinian Energy Within the Middle School Curriculum

To develop and implement Einsteinian energy educational content for middle school, we had to consider students’ zone of proximal development at the Year 8 level. The current middle school curriculum in Australia and many other countries include outdated physics concepts. For example, mass conservation and the Bohr model of an atom are valid approximations within the limits of the description of scientific phenomena. However, according to modern science, they provide a distorted representation of phenomena. Thus, modifying the curriculum is required if we want this new Einsteinian physics knowledge to be part of teachers’ and students’ learning (Tytler & Symington, 2006). It is worth mentioning that many modern topics in the physical, biological and chemical sciences are already part of the middle school curriculum (e.g. DNA replication, the big bang and photosynthesis).

Educators are revisiting essential physical science topics that are recommended to be part of the middle school curriculum (Boyle, 2019; Kersting & Blair, 2021; Kneubil, 2019; Lacy et al., 2022; Pietrocola & Gurgel, 2017; Preston et al., 2020). Including Einsteinian physics in middle school provides a solid basis for students to understand the relationship between physics and technology (Kersting & Blair, 2021). The introduction of Einsteinian energy links disparate energy processes—including nuclear, quantum, chemical, gravitational, light, heat and electricity—and provides a simplified conceptual framework for understanding energy relevant to many areas of science (Boublil & Blair, 2023).

Einsteinian physics has been and continues to be the framework for understanding modern discoveries (Kersting & Blair, 2021). It contains the foundational concepts of biology, chemistry, engineering, technology and physics. Introducing Einsteinian physics in the middle school curriculum allows us to develop a curriculum in which the historical discovery of energy is connected to Einsteinian energy and traditional Newtonian concepts. The current Australian curriculum (ACARA, 2021) includes a strand called “Teaching science as a human endeavour”, which emphasises the notion that scientific knowledge changes over time. Portraying the evolution between Newtonian (classical) and Einsteinian physics (Velentzas & Halkia, 2013; Villani & Arruda, 1998) highlights how and why scientific knowledge changes over time.

The module described in this research paper connects concepts already part of the Australian curriculum to the modern understanding of energy. It facilitates the integration of new content relevant to a better understanding of energy. At the middle school level, the Australian curriculum emphasises the importance of learning energy and that students need to “develop their understanding of microscopic and atomic structures; how systems at a range of scales are shaped by flows of energy and matter and interactions due to forces, and develop the ability to quantify changes and relative amounts” (ACARA, 2021). In the Year 8 chemical science strand, students explore the “changes in matter at a particle level and chemical and physical change”. In the physical science strand, students “begin to classify different forms of energy and describe the role of energy in causing change in systems (kinetic and potential energy), including the role of heat” in energy transformations. Modern topics of energy, which are part of Einsteinian physics, are not included in the Australian science curriculum for the middle school level (Years 7, 8, 9 and 10).

Concepts inherent in Einsteinian energy are essential. Learning about photons provides a framework for understanding heat, the electromagnetic spectrum and energy transformations. The units of energy and power (joules and watts), crucial to the equations of Einsteinian energy, facilitate the students’ understanding of kinetic energy, potential energy, heat energy and energy conservation and transformation. At the Year 9 level, students explore the “wave and particle models for light; heat in terms of convection, conduction and radiation; energy through an electric circuit; and properties of waves using light and sound”. The equations *E* = *mc*^2^ and *E* = *hf* provide a natural energy framework for both year levels, emphasising how these equations are tied to all energy-related topics covered in the Australian curriculum. Einsteinian energy concepts allow a broad range of processes to be unified under the concept that energy has mass. These processes range from nuclear fusion and fission to DNA replication and from photosynthesis to black hole coalescence.

## Research Context

### The Einstein-First Project

The Einstein-First project is an Australian educational research project that focuses on the design of teaching and learning Einsteinian physics in schools (Kersting & Blair, 2021). The Einstein-First project emphasises the need to learn modern physics concepts in schools, and the team’s researchers are dedicated to developing novel ways to engage students to learn through the use of hands-on activities (Choudhary et al., 2018; Kaur et al., 2017a, b).

The first intervention of Einstein-First was conducted with Year 6 students (aged 10–11) at an Australian primary school in 2012 (Adams et al., 2021). It comprised six 45-min lessons, a reading play and an excursion to the Gravity Discovery Centre, located just outside Perth. This first exploratory research by Pitts et al. (2014) investigated the impact of an enrichment program based on selected Einsteinian physics concepts. They analysed students’ pre- and post-physics knowledge and attitudes. The results showed promising benefits, highlighting the importance of teaching Einstein physics to young students. Researchers at the Einstein-First project have conducted multiple interventions in Australian schools since. Short- and long-term interventions and follow-up questionnaires observed significant positive results in students’ appreciation and understanding of Einsteinian physics (Choudhary et al., 2018, 2020; Pitts et al., 2014). The Einstein-First project is now developing a structured Years 3–10 curriculum tailored for teachers desirous of introducing Einsteinian physics concepts in their classrooms (Kersting & Blair, 2021).

### Challenges in Teaching Einsteinian Physics and Energy

More research is needed on teaching energy in school. Researchers are still testing theories and methods to understand better what and how to teach specific energy topics (Bächtold & Munier, 2019; Constantinou & Papadouris, 2012; DeWaters et al., 2013; Kubsch et al., 2020). There is a lack of consensus in the literature when considering what exact topics should be introduced at the secondary level. For example, secondary students have shown difficulty understanding energy abstractly in processes like heat, electricity and light (Constantinou & Papadouris, 2012). They also tend to confuse energy with concepts such as force, power and heat. Energy is vital to our modern society, and students should learn what it is and how to appreciate it (Bächtold & Munier, 2019; Constantinou & Papadouris, 2012; DeWaters et al., 2013; Duit, 1984).

Research on teaching modern physics energy topics in lower secondary schooling is new. The energy-mass equivalence presents many epistemological obstacles; the didactic intentions become more complex as the teacher must discuss contemporary and innovative related topics because modern experiments are beyond their reach (Kneubil, 2020). For example, the energy of a proton in the large hadron collider must consider its mass increase with speed, and this obviously cannot be done in the classroom.

One of the main challenges in teaching energy to school students lies in developing strategies to demonstrate the intricacies inherent in energy conservation (Bächtold & Munier, 2019). This concept is important because students need to consider it when working with the equation *E* = *mc*^2^. Hands-on qualitative experiments that use analogies and activities are essential for students to understand various concepts in Einsteinian physics (Gingras, 2015; Kaur et al., 2017b; Kersting et al., 2018; Treagust et al., 2002). Introducing quantitative experiments to test Einsteinian physics is more appropriate for upper-secondary and university-level physics.

## Aims and Research Questions

This paper covers the various phases of designing and evaluating a Year 8 Einsteinian physics energy teaching module as part of the Einstein-First project. The aims are to:

1. Identify and reconstruct appropriate level key core concepts of Einsteinian energy
2. Develop a teaching module that includes key core concepts of Einsteinian energy
3. Evaluate the module in terms of students’ understanding of core concepts

The learning module contains core quantum physics and relativity concepts designed for teachers and their students. Analysis of the first cycle of the development of this curriculum is designed to provide (1) an overview of theoretical perspectives associated with the concepts involved; (2) results of an initial study; and (3) a deeper insight into students’ learning. The subsequent research question that drove the study are:

- R1. What education design principles should we adopt to develop a teaching–learning sequence?
- R2. What are the students’ pre-instructional and post-instructional understandings of the concepts involved?
- R3. Were the learning goals achieved?
- R4. What educational design principles should we develop for future trials?

## Theoretical and Methodological Framework

We have used two complementing educational design frameworks—*design-based research* (Reeves, 2005) and *the Model of Educational Reconstruction* (Duit et al., 2012). These two methodological frameworks illustrate our approach to designing and evaluating the learning environment.

### Model of Educational Reconstruction and Design-Based Research

The Model of Educational Reconstruction (MER) is a methodological framework that provides an instructional guide for improving teaching practices. Our primary goals within this framework are to provide (1) clarification and analysis of the science subject, (2) investigation of students’ and teachers’ perspectives of the science subject and (3) design and evaluation of learning environments (Duit et al., 2012). The framework provides guidelines for scrutinising the educational relevance of Einsteinian physics, a science field that has not yet entered mainstream education (Kersting et al., 2018).

The design-based approach is useful as it provides a structure for addressing questions outlined in the research statement (Canu & Duque, 2014; McKenney & Reeves, 2013a). Adopting this systematic approach helps develop and implement solutions to our specific educational challenges. The designed-based approach outlines four main phases in educational research:

#### Phase 1: Preliminary Analyses and Design of the Learning Environment

Einsteinian physics concepts are first identified and described, connecting them to current physics content in the Year 8 Australian Curriculum. Framing and designing a learning module will allow us to assess better the steps needed to develop a coherent learning structure. We will then provide an epistemological analysis of content and an analysis of teaching. Equally important is a description and analysis of students’ conceptions, difficulties and obstacles that influence their development in learning the topics covered in the corresponding years.

#### Phase 2: A Priori Analysis and Design of the Teaching Situations

The choice of content (physics and teaching approach) is chosen to help construct the didactic situation for teaching the physical concepts of Einsteinian energy. Theories of conceptual change aided in determining how student conceptions develop (Dykstra et al., 1992; Treagust et al., 2002; Vosniadou, 1994). We also used the *theory of didactic situations* (Brousseau, 2002), a framework for building teaching sequences that target specific physical problems that need to be solved. It also includes tasks guiding students and teachers to question scientific phenomena. Questioning becomes a central part of the inquiry process, leading to acquiring new knowledge (Brousseau, 2002). Previous positive research outcomes of the Einstein-First project encouraged us to use active learning methods based on qualitative and quantitative use of analogies. This approach fosters the visualisation of abstract concepts, thought experiments, group discussions and historical and philosophical perspectives.

#### Phase 3: Iterative Cycles of Testing, Refinement of Solutions in Practice

In this phase, the content is made accessible to the teachers, and they become co-investigators in the research, whereby using a collaborative approach (Voogt et al., 2015), the teachers evaluate and improve the Einsteinian physics content. Questionnaires and interviews with individual teachers were used to collect data for in-depth analysis. This stage also includes observation protocol and audio recording of teaching sequences when necessary. Data from teachers were analysed with a specific focus on teacher perspectives of learning resources/activities, lessons and confidence to teach content and concepts.

#### Phase 4: Post-analysis and Evaluation

In this phase, data from students’ responses to the questionnaire are analysed with a focus on the relevant content from the Einsteinian physics Year 8 module. A pre-/post-test assessment tool was used to help us determine students’ conceptual change. In addition, the thematic analysis of students’ responses to the tests allowed us to assess students’ conceptions better. The conclusions drawn from these analyses helped improve our formative assessment of the teaching module.

### Student Sample

We conducted the study in an independent state government school in Western Australia for 4 weeks as part of a sequence of nine core lessons on Einsteinian energy. The research work was implemented with a science specialist teacher and their Year 8 classes (13–14 years old), 22 students in total. During in-class observations, we identified the weaknesses and strengths of the selected series of lessons from the Year 8 curriculum based on the collaboration between the teacher and researcher.

### Data Collection and Analysis Methods

The design of this study followed an exploratory case study model (Yin, 2017). The Year 8 energy program consisted of nine lessons focused on activities, presentations and worksheets. We used mixed methods to explore further our understanding of the program (Creswell & Creswell, 2018). The first author of this paper acted as a developer, teacher trainer, co-teacher, collaborator and observer throughout the trial. The teacher who took part in the study is not considered a “research subject” but a research collaborator willing to engage in implementing the Einsteinian physics curriculum. This collaborative approach was vital from the outset when interacting with the teacher.

#### Student Test

This paper focuses primarily on students’ results from the questionnaires, not the observational strategies and the teacher interviews. We administered a knowledge pre- and post-test with 15 identical questions that included open-ended and multiple-choice questions targeting students’ understanding of the central concepts of the learning goals and fundamental features of the Year 8 module on Einsteinian energy. We developed a marking rubric with a total score of 15 points for the pre-test and post-test to analyse students’ scores.

### Analysis Procedures

We categorise the blank responses in the results, as not all students responded to all items. The questionnaire covered pre-conceptions along with core concepts central to our teaching module. We graded students’ responses with an increasing value, up to one point for each item. Student responses to individual test items (questions) gave us an insight into students’ understanding of physical concepts. We entered the scores in excel, a suitable software for our purpose. The paired samples *t*-test was used to determine if there was any statistical difference in the students’ mean scores over the program period. Cohen’s *d* effect size was calculated via the guidelines proposed by Cohen (1988, pp. 284–287).

Developing questions that reflect modern physics energy topics at the Year 8 level was challenging. The first author initially drafted the test questions and then discussed them with the other authors and Einstein-First project team members. We referred to university-level physics textbooks and middle school energy examinations to find suitable question structures that could be modified for the topics in our curriculum. The final questionnaire consisted of items that focused on students’ understanding of the Einsteinian energy topics highlighted in the learning goals in Table 1.

**Table 1:** An overview of the classroom lessons conducted by the teacher and researcher

Lesson number and topic	Content and learning intention	Pedagogical approach
1. Introduction to Einsteinian physics energy	- Understand the difference between energy and power, when using Joules and Watts - General introduction to *E* = *mc*^2^ and *E* = *hf* and its connection to real world - Investigate the conservation of energy	- Group brainstorm about energy - Calculation activity for understanding joules and watts - Activities for doing different forms of energy. Using your stored chemical energy to move
2. The powers of ten and the speed of light	- Use the speed of light to measure distances in our universe - Understand that photons travel at the speed of light in space - Understand how to multiply and divide using the powers of ten	- Measure the speed of a nerf gun bullet - Problem-solving situations allowing students to understand the connection between distance, time and speed: distance between the moon and the Earth with light and the mirrors from the Apollo 11 mission, the value of a light year, travelling to our nearest star (Proxima Centauri) with the project Starshot, worksheet exercises to measure distances in our universe, e.g. the distance between our sun and the centre of our galaxy
3. The discovery of photons	- Observe or role play a historical interpretation of Einstein’s and Planck’s discovery of photons - Qualitative understanding of Planck’s constant - Understand that photons have energy and carry momentum like bullets	- Acting in a scripted play where students get to perform different characters and talk about the discovery of photons - A nerf gun photo electric effect competition - Using M&Ms to understand distribution
4. Waviness and bulletiness of photons	- Appreciate a historical background of Einstein’s and Planck discovery of photons - Understand that photons have energy and carry momentum like bullets - Understand that photons can impart their energy to an electron bound to an atom - Understand the concept of frequency and its tie to photons - Understand that photons can create interference patterns	- Students will use a worksheet to identify where they experience the concept of bulletiness and waviness of photons in each of the 6 activity stations: (1) nerf gun photons ejecting ping-pong electrons from a bowl; (2) shining different lasers on phosphorescence paper; (3) shining laser on a human hair to observe interference and diffraction; (4) shining a laser on soap film to observe interference and reflection; (5) using a slinky to relate to the connection between energy and frequency; (6) using a mini solar car to observe how light can impart energy
5. Understanding the photon spectrum with *E* = *hf*	- Calculate the energy and frequency of different photons - Use energy and power units (joules and watts)	- Using *E* = *hf* to understand the whole electromagnetic spectrum using the worksheet - Calculating how many photons are present in 1 J of energy
6. How physicists measure *E* = *mc*^2^ in the lab	- Use *E* = *mc*^2^ formula - Use energy and power units (joules and watts) - Understand the relationship between *E* = *mc*^2^ and *E* = *hf* - Describe how energy has mass	- Using a mass attached to ruler to understand inertia and how mass is measured - Using magnets to suspend a pencil - Shining different lasers on phosphorescence paper to understand how atoms can absorb and release photons of certain wavelength
7. Phasors and interference	- Use the phasor wheel to understand frequency, wavelength and speed - Demonstrate how vectors are added - Demonstrate how the phasor wheel can allow us to understand the double-slit experiment	- Using 5 different sized phasor wheels to understand how a spinning wheel can relate to frequency, wavelength and speed - Worksheet and exercise on adding vectors - Exercise on using vector addition and the path of a phasor wheel to understand the concept of photon interference and probability
8. Binding energy	- Understand how nuclear energy is produced and how energy is conserved - Understanding that binding energy is the energy that holds bonds and the nucleus of an atom together - Use diagrams to explain the difference between fission and fusion reactions - Connect different activities to the concept of *e* = *mc*^2^ and *e* = *hf*	- Use magnetic tennis balls and buzzing magnets to make analogies between magnetic binding energy and nuclear binding energy - Using a video and an online app to look at the concept of binding energy and fusion. Focus on how energy is produced in the sun - Using a video and calculation activities to look at the mass deficit of chemical and fission reactions
9. Gravitational waves and binding energy	- Understand black holes mergers - Explain how Einstein’s theory of gravity is used to describe black holes - Use *E* = *mc*^2^ to determine how much mass is converted into gravitational wave energy in black hole mergers	- PowerPoint on black holes - Videos about black hole detection and Ligo - Use the Sci Vr app to look at black hole mergers and curved space - Using Ligo’s poster of 2021 to calculate the energy release in 3 events of your choice

## Construction of an Einsteinian Energy Module

According to the MER, constructing a new educational curriculum requires analysing and synthesising core physics concepts to determine learning goals. Educational researchers attribute the role of experience and reasoning when building new knowledge (Anderson & Wall, 2016; Bancong & Song, 2020; Bungum et al., 2015, 2018; Steier & Kersting, 2019). There are also elements such as doubt, creativity and imagination seen by many physicists as necessary for constructing Einsteinian concepts and developing scientific thinking (Bachelard, 1957; Steier & Kersting, 2019).

### Key Concepts Associated with Einsteinian Energy

To identify core concepts and gain more perspectives on how educators and researchers treat these concepts, we referred to a list of research papers that cover energy topics in quantum and relativity (Baierlein, 2007; Bungum et al., 2015; Henriksen et al., 2014; Ireson, 2000; Kaur et al., 2017b; Kneubil, 2019, 2020; Otero et al., 2015). The above articles adopted various approaches to teaching quantum physics, ranging from online environments, hands-on activities, and mathematical problem-solving to philosophical considerations. The mentioned articles cover the particle nature of light, the wave-particle duality, the energy-mass equivalence, the photon momentum, the photo-electric effect, the Heisenberg indeterminate principle and more. However, this article deals for the first time with the relationship between *E* = *hf* and *E* = *mc*^2^ and their relevance to learning about energy and modern physics in schools. There are different methods for teaching the fundamentals of quantum physics, but there is no consensus on what and how to teach it in high school. Therefore, we had to define the intelligibility of concepts for our target year level. We referred to educational research, modern physics research, the Australian curriculum and the creative nature of developing resources to reconstruct energy and quantum at the Year 8 level. Our proposed module is based on these two equations, *E* = *mc*^2^ and *E* = *hf*, since they best describe all the energy processes in the universe. One describes how energy has mass, and the other describes how light and heat consist of tiny packets of energy called photons and phonons.

**Fig. 1:**
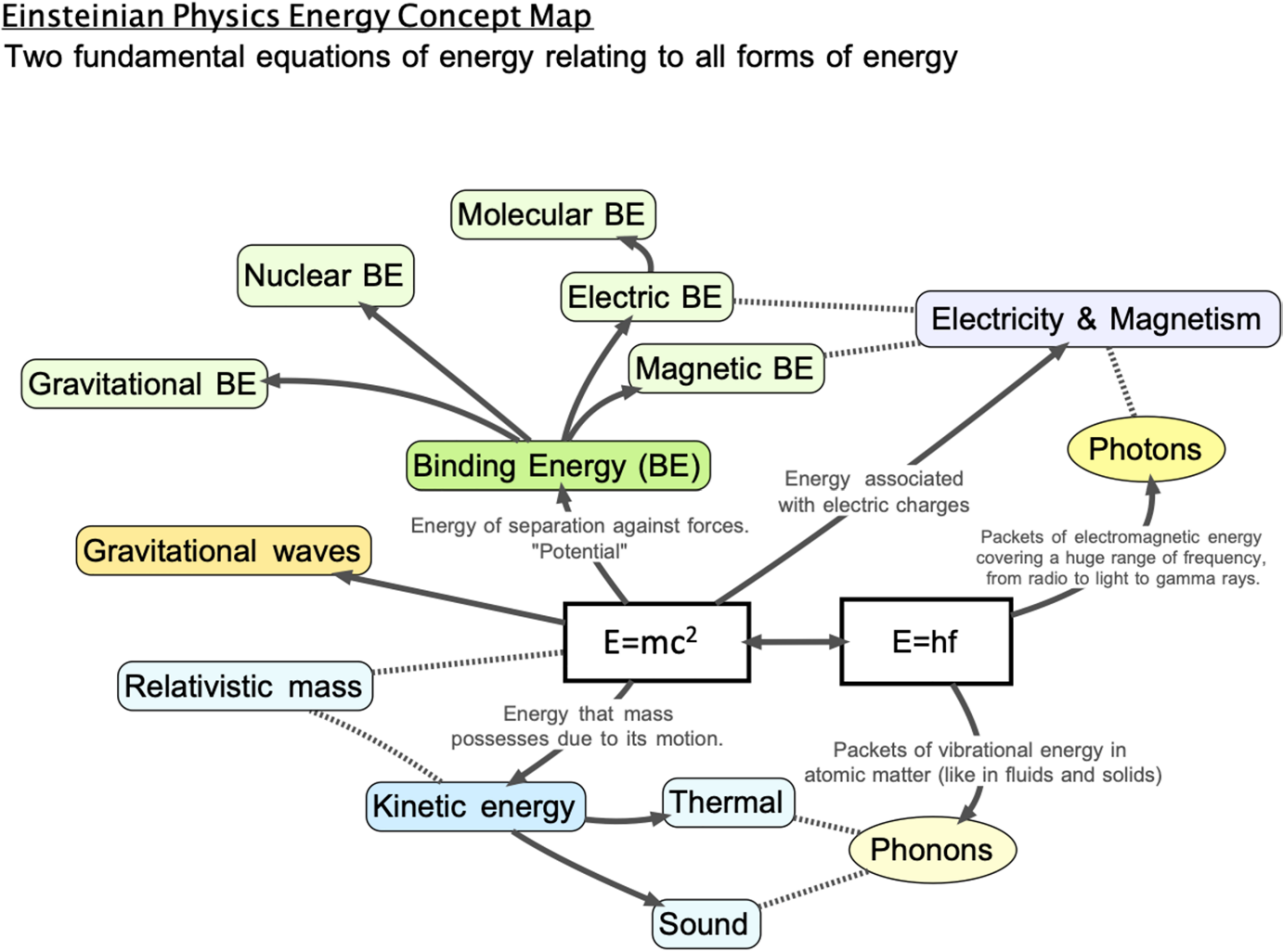
Einsteinian energy concept map. Two fundamental equations of energy relate to all forms of energy. The concept map was designed by Shachar Boublil and David Blair

The concept map in Fig. 1 represents our modern view of energy using these two Einsteinian equations. Both provide us with a framework for understanding all forms of energy. They either fit in the category of *E* = *mc*^2^ or *E* = *hf*. The equations help describe how a photon can impart energy to an object with inertial mass, like an atom, and give it more mass. Recently, researchers measured this change in the inertia of an atom when brought to an excited state (Schüssler et al., 2020). This experiment is an excellent example of how energy is conserved and transformed: where the total energy of a system (including mass) can be converted from one form to another on a scale that is unperceivable to the human senses. Our article shows how we can use hands-on experiments and mathematical problem-solving to understand the fundamental concepts of this experiment (Boublil & Blair, 2023). These two equations are simple equations of proportionality, like *y* = 6*x*. These equations of proportionality have very large and small constants: in *E* = *mc*^2^, the constant *c*^2^ is the speed of light times itself (roughly 9 × 10^16^ m^2^/s^2^); in *E* = *hf*, the constant *h* is Planck’s quantum constant which is roughly 6.6 × 10^−34^ J/hertz, and it says that the energy of a photon depends only on its frequency *f*. For students to understand the equations, they need to know how to manipulate the powers of ten. The extremely small magnitude of the universal quantum constant is connected to our understanding of photons that impact the surface of our skin, generate power with solar panels and determine temperature. We have identified learning intentions and used them in developing activities and experiments for the series of lessons presented in Table 1.

## Principles in Building Teaching–Learning Sequence for Einsteinian Physics

Building design principles for Einsteinian physics concepts is part of developing a new curriculum. Integrating the design principles in the development of lessons is a way to “develop solutions to practical problems in learning environments” (Reeves, 2005, p. 76). The principles developed by the different research groups in teaching Einsteinian physics (Baierlein, 2007; Bungum et al., 2015; Henriksen et al., 2014; Ireson, 2000; Kaur et al., 2017b; Kneubil, 2019, 2020; Otero et al., 2015) are based on the following factors: identification of core concepts, educational approaches and models used for teaching, analysis of students’ conceptions and analysis of learning obstacles. The design principles developed by these research groups include methods that promote a qualitative understanding of modern physics. These are thought experiments, analogies and visualisations of quantum phenomena; group discussion of the coexistence of different contradictory theories; writing workshops; reflections on historical and philosophical perspectives of science; presentation of examples of resolved scientific conflicts; and examples of unresolved scientific conflicts.

We have developed the following general principles to be applied in the design and implementation of teaching–learning sequences: (1) provide examples linking formulas to real-world scenarios; (2) provide visualisation techniques, simulations and analogical activities to highlight ideas; (3) encourage discussions and collaboration; (4) emphasise the need to understand mathematical symbols and their meanings; (5) focus on how physics relates to modern-day technologies; (6) focus on how the powers of ten are used to understand Einsteinian energy; (7) emphasise the importance of the history of science.

Our research goal is to evaluate our environment and students’ performances to develop our design principles further. Deriving design principles in the analysis of our first study inform the future development of our curriculum. We should not forget that “Design principles are not intended as recipes for success, but to help others select and apply the most appropriate substantive and procedural knowledge for specific design and development tasks in their own settings” (McKenney & Reeves, 2013b, p. 110). The table in the appendix summarises lesson content, learning goals and pedagogical approaches. We used hands-on activities and mathematical problem-solving to present core concepts crucial to this curriculum.

## Results

**Fig. 2:**
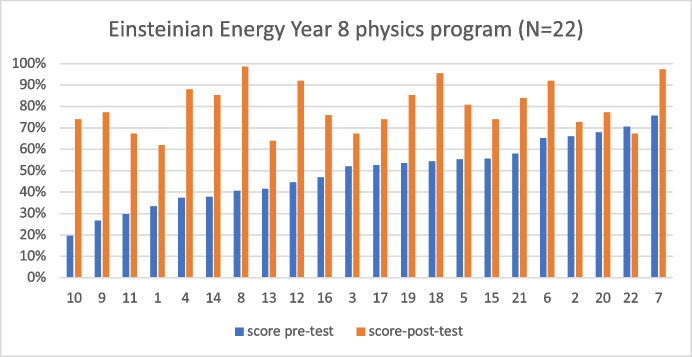
Pre-/post-questionnaire results of 22 students from Year 8 in the program. Based on the results from the pre-test, we arranged students’ results from the lowest score to the highest score. The 100% represents a score of 15

We categorise students’ results from the pre-/post-questionnaire in ascending order. Figure 2 shows that 21 students improved their scores over the trial period, and only one student’s score did not improve. The pre-test questionnaire comprises 15 questions that are identical to the post-questionnaire. The scores are given as percentages in Fig. 2, where the maximum score of 100% represent 15 points.

A paired-samples *t*-test was taken from the pre-/post-test results, showing the statistical significance of the pre-test score (*M* = 7.40, SD = 2.25) to the post-test score (*M* = 11.95, SD = 1.66), where *t*(22) = 7.62 and *p* < 0.001 (two-tailed). The mean increase is 4.55. Cohen’s *d* is 1.98, and the confidence interval of 95% ranges from 1.24 to 2.69. Figure 2 shows that most students improved their conceptual understanding of core concepts. Students that had a lower and medium initial score showed dramatic improvement. Here, we focused on core concepts central to this new curriculum which investigates students’ conceptual understanding of energy using *E* = *mc*^2^ and *E* = hf. The most significant improvements were observed within the first group of students with initial low-test scores; for example, students 10 and 8 had improvements of about 55% and 58%. In contrast, student 7, with an initial score of 76%, increased their score to 97%. This shows that students with different initial understandings can develop and demonstrate improvements to attain similar scores in understanding Einsteinian physics and energy topics. The student that did not show score improvement is due primarily to the delay in administering the post-test concerning the content covered in the lessons. We administered the post-test after students had covered a 4-week electricity module on energy. The student also showed signs of guessing correct answers in the multiple-choice questions in the pre-test.

In the following two sub-sections, we focus on questions relating to (1) the energy-mass equivalence and (2) photons. Students’ responses will reflect their understanding of concepts and content introduced in the lessons. Responses highlighted below are before and after the module (Table 2).

**Table 2:** Questions and results. The pre- and post-test scores are on the table’s right-hand side

Questions and pre- and post-test results from the Einsteinian energy program		
Questions	Pre-test	Post-test
1. Choose one of four statements that best describes the equation *E* = *mc*^2^ and explain	41%	86%
2. Choose one of four statements that best describes the equation *E* = *hf* and explain	18%	68%
3. Light travels at 3 × 10^8^ m/s (300 million meters per second). Assume, that the moon is roughly 3 × 10^8^ m away. How much time does it take light to get from the moon to Earth?	82%	93%
4. Light travels at 3 × 10^8^ m per second. The sun is roughly 8 min at light speed away. Roughly, how far away is the sun from earth in meters? (show your calculations)	59%	68%
5. Match the quantity to the unit in the following table: (1)Energy, (2) time, (3) power, (4) distance, (5) mass	91%	100%
6. What is the main form of energy for each of the following situations? Associate the right answers	69%	95%
7. You are watching a YouTube clip on your phone. Choose the best description of the energy transformations that are happening	55%	100%
8 Modern phones take around 1 h to charge (60 min, 3600 s). If your phone uses 10 J per second when charging. How many joules do you need to charge your phone?	77%	82%
9. If you weighed an object on a supersensitive balance, would the balance register a different weight if you heated the object up? Tick Yes or No and explain your answer	68%	86%
10. The power output of the sun each second is joules energy. Using calculate the mass quantity lost by the sun	0%	43%
11 Associate these physical phenomena to these different categories of energies: (1) Kinetic energy, (2) heat energy, (3) binding energy, (4) nuclear energy, (5) magnetic energy	53%	63%
12. Which photons in the solar electromagnetic radiation spectrum damage our skin and destroy the DNA structure of our cells?	32%	73%
13 Complete the following sentences, by using the words from the list below	55%	100%
14. This diagram shows a solar cell that is used to operate a solar fan Complete the following energy flow diagram. Order these different forms of energy in the energy flow diagram. Electric energy, solar energy, mechanical energy, magnetic energy	27%	59%
15. Calculate the Energy in joules of an ultraviolet photon of frequency using *E* = *hf*, where Planck’s constant *h* = 7 × 10^–34^ J/Hz	14%	77%
Mean	49%	80%

### Energy Has Mass Questions—We Focus on Three Questions

*Question 1 students’ general understanding of E* = *mc*^*2*^*.* In the pre-test, 9/22 students answered correctly, selecting the statement “energy has mass”. Out of these answers, five students provided an explanation that described the two of the symbols used in the equation, as for student 2: “*E* represents energy and m represents mass”. Two students did not answer, and one student wrote that “energy takes up space”. Seven students chose the “energy of a photon” without a coherent explanation. Six students chose “the speed of light is a constant” and gave incoherent explanations. In the post-test, 19/22 students chose the correct answer: energy has mass. Out of these answers, nine students provided an explanation that described all the symbols used in the equation. Here is an example of a clear and acceptable explanation: *Student 2:* “*E* = *mc*^2^ means energy has mass. It means *E* (energy) is equalled to the mass (*m*) times the constant of light at (3 × 10^8^)^2^”. Two students gave a good explanation detailing their conceptual understanding of the equation.

Student 4: When the energy in an object changes, its mass changes. This is how we know that energy has mass.

Student 11: energy having mass is proven when a flashlight was shined, then later weighted and turned out to be lighter.

Four students gave an incomplete explanation, highlighting mathematically that energy equals mass. We associate this with the energy-mass equivalence. Student 15 was the only one to choose the “energy of a photon” and gave a coherent explanation to support his answer, “The way to calculate the energy of the photon is by multiplying the weight by the speed of light squared”. This was also the only student that referred to a photon carrying inertia. This answer directly resulted from our lesson on teaching that a photon can impart mass to an atom. We observed that 2/22 students chose “The mass of an object is equal to its energy”, and only one student gave a correct answer with no coherent explanation to support it.

*Question 9* “If you weigh an object on a supersensitive balance, would the balance register a different weight if you heated the object up? Tick Yes or No. Please give a reason for your answer.” Before the teaching module, 15/22 students responded “yes”. Two students used *E* = *mc*^2^ and energy to support their answers. Six students used the concept of heat and expansion but did not show an increase or decrease in weight. Here is an example: *student 2:* “yes, when things are heated, the particles become rapid, and the mass expands”.

Five students wrote that the object would weigh more. One student wrote that there would be a decrease in mass, and one student gave no answer. 7/22 students responded no to question 9. Three students were unsure. Three students wrote, “when an object is heated, the particles expand, increasing the volume and overall density. This, however, wouldn’t change its mass”*.* One student wrote, “you are not adding anything to the object”.

The post-test revealed that 19/22 students responded yes to question 10. Thirteen students used *E* = *mc*^2^ and energy having mass to support their answer. Here is an example: *student 4:* “Yes. Because Einstein discovered that energy had mass and is a form of energy”.

Three students used the concept of heat and expansion but did not indicate the increase or decrease of weight. This misconception is associated with the relationship between mass and volume. Three students did not answer, and the students that responded no to question 9 (3/22) did not explain their choice.

*Question 10* “The power output of the sun is $4 \times {10}^{26}$ joules of energy each second. Using $E=m{c}^{2},$ calculate the mass quantity lost by the sun each second”. Prior to the program, students could not solve question 10. After the program, the average class score was 9.25/22. One student understood the mathematical logic of the question but forgot to square the speed of light and how to divide using the powers of 10. *Student 14:* “*m* = *E*/*c* = 4 $\times$ 10^26^ Joules/3 $\times$ 10^8^ = 1.33 $\times$ 10^34^”.

Nine students showed they could use the powers of ten and the logic required to solve the problem using the *E* = *mc*^2^ equation. Students had the most difficulty with this question because it involved dividing using the powers of ten and multiplying the speed of light constant. This is primarily due to only two lessons covering the mathematics and concepts involved in understanding the energy-mass equivalence equation. It is worth noting that all students solved similar problems in the lesson 6 worksheet we administered and used the *E* = *mc*^2^ equation to only solve for the gravitational wave energy of black hole mergers in lesson 9.

### The Energy of Photons—We Focus on Three Questions

Question 2 allowed us to gain insight into students’ thinking about *E* = *hf* connecting the energy of a photon before and after the teaching module. Before the teaching module, 4/22 students chose the correct answer: “Energy of a photon” best describes the equation. Only one student described the symbols used in the equation, “energy, frequency and Planck’s constant”. Another student referred to the last question in the test, which is on *E* = *hf*, and two students did not provide any explanation. 16/22 students chose “energy has frequency” and explained that *E* is energy and *hf* is for high frequency. Also, 2/22 chose “the speed of light is a constant” and gave no coherent explanation for their answer. None of the explanations identified the symbols or described the equations conceptually. After the teaching module, 15/22 students chose the correct answer, “energy of a photon”. Out of these answers, eleven students provided an explanation that described the symbols used in the equation. Here are two good examples:

Student 5: *E* = *hf* means the energy of a photon, a particle of light. It means *E* energy is equalled to Planck’s constant (7 $\times$ 10^–34^) times the frequency of a photon.

Student 17: The reason I chose this answer is that *E* = *hf* stands for energy = Planks constant (7 $\times$ 10^–34^) x frequency. We know energy has a frequency since light (form of energy) contains tiny particles that make up light (photons) and light moves in a wavelike motion.

There were (6/22) students that chose “energy has high frequency. Only one student gave an elaborate and Einsteinian explanation: *Student 19:* “e = hf means that Planck’s constant multiplied by the frequency of the electrons equals the amount of energy of electrons”.

Question 12 allowed us to validate if students remembered which solar photons would damage the DNA structure of our cells. Before the program, 7/22 students wrote that ultraviolet light would destroy the DNA structure of our cells. After the program, 16/22 students achieved the correct answer. Six students wrote gamma photons, and ten students wrote ultraviolet photons.

**Fig. 3:**
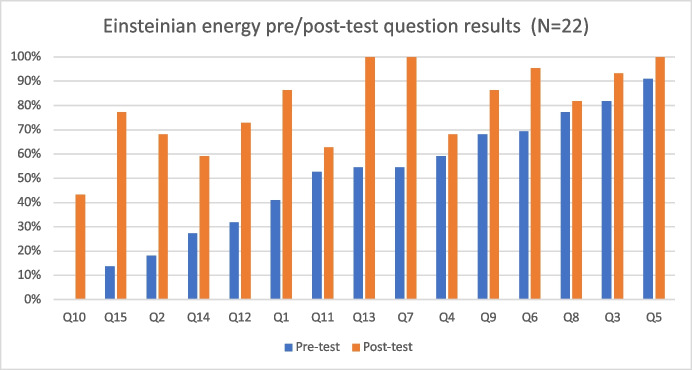
Students’ pre-/post-test mean score for each question. The results show that students improved their conceptual understanding of every concept after the Year 8 energy program. We arranged the questions from the most difficult to the least difficult based on the pre-test results

Question 15 asked students to calculate the energy of an ultraviolet photon of frequency 10^16^ Hertz using *E* = *hf*. Before the program, 3/22 students understood how to use the powers of ten and this equation to find the energy of a photon. After the program, 17/22 students knew how to calculate the energy of a photon using the powers of ten. Students’ results to this question showed the most improvement. The total score difference between the pre-test and post-test is wider than in any other question (see Fig. 3).

The pre-/post-test results in Fig. 2 show that scores improved mainly from students that initially had medium and lower total scores. This suggests that the enrichment program improved the results for most students. Only one student showed a lower score in the post-test. Obviously, there is always room for improvement as many factors can influence these results like testing methods, the efficacy of lessons, delay in administering the post-test and students’ motivation.

## Discussion

The findings from this study, involving 22 Year 8 students in one school in Australia, showed significant improvements in students’ understanding of core Einsteinian physics concepts. Data from questions 1 and 2 of the questionnaire on the equations *E* = *mc*^2^ and *E* = *hf* show that more than half of the students in the class responded correctly, choosing appropriate statements relating to the equations. Approximately half of the students consistently used correct interpretations of Einsteinian physics. Written explanations given by the other half needed improvement. Conceptually most students understood that energy has mass, and that light comes in photons. They also showed a good understanding of using the powers of ten when finding the energy of a photon. However, students showed the most difficulty in measuring the mass loss using the *E* = *mc*^2^ equation. We also observed improvement in students understanding of using the powers of ten and categorising forms of energy and energy transformations.

Our findings from this study have led us to identify the challenges in understanding Einsteinian energy at the Year 8 level. This analysis has allowed us to formulate empirically based research principles, which will help implement future project resources and trials. We have revised our design principles, adding them to our resources. We outline these principles below in a two-level hierarchical structure (Table 3).

**Table 3:** Design principles for learning resources for Einsteinian energy in Year 8

1. Link key mathematical concepts of Einsteinian physics to real-world scenarios: - Show how *E* = *hf* relates to the whole photon electromagnetic spectrum - Show how *E* = *mc*^2^ and *E* = *hf* relate to the experiment in finding the mass of an atom and to the energy released from the sun - Use *E* = *mc*^2^ to determine the mass lost by the sun each second and the energy released from gravitational waves - Use *E* = *hf* to determine the number of photons emitted by the sun each second - Use the speed of light to measure distances in our universe, like the distance between our Sun and Proxima Centauri with the project Starshot and the distance between the earth and the moon with the mirrors left on the Apollo 11 mission
2. Link key concepts of EE to real-world scenarios using analogies and activities: providing visualisations techniques, simulations and analogical activities to highlight relevant images to the curriculum: - Use lasers to look at interference patterns (hair, double slit, soap film), absorption and emission of photons from phosphorescence paper - Show how a pulse of light (photons) can be emitted and visualised using Femto-photography - Show how single photons can interfere in the double-slit experiment - Using classical physics to understand modern experiments: nerf gun/ping-pong photoelectric effect, slinky to understand frequency, phasor wheels to understand frequency, wavelength and speed, magnets to look at binding energy, using inertia to measure mass with a flexible ruler, suspend and trap an object using magnets - Using app simulations to visualise quantum phenomena, binding energy and relativistic phenomena
3. Focus on the mathematical complexity of equations: - Use the powers of ten to understand the concept of energy, frequency, Planck’s constant, the speed of light, mass, distance, time and quantity - Rounding up numbers to be approximately right makes working with the powers of 10 easier - Provide a framework for students to understand how symbols connect to numbers and physics - Provide simple tasks and examples to initiate and motivate students to divide and multiply using the powers of ten - Emphasise the importance and meaning of constants in equations
4. Focus on the history and the nature of scientific knowledge: - Encourage students to question and work in groups when discussing Einsteinian physics - Include drama-role plays to look at historical scientific events and physical phenomena - Present authentic interpretations of scientists and their ideas to the target audience - Provide connections to experiments from the past, present and future

## Conclusion

In this study, we described the development and implementation of a Year 8 program on Einsteinian energy. Following the design-based research and MER framework, we identified learning goals addressed in the lessons we developed and the pre-/post-test we administered. Subsequently, we tested the program to understand the challenges and develop new educational principles. The classes result from the pre-/post-test indicated a 31% mean increase. Our study contributes to understanding the challenges in learning Einsteinian physics and energy. We presented empirical results of middle school students’ understanding of *E* = *mc*^2^ and *E* = *hf*.

In summary, our results support the idea that middle school students can acquire a qualitative and quantitative understanding of Einsteinian physics. To teach this content, we must link concepts to appropriately designed lessons that are engaging for students and teachers. We do this using hands-on activities, presentations, videos and engaging worksheets. This teaching module may need to be adapted for different classrooms and schools. In our case, we carefully integrated the two equations into the framework of the existing curriculum on energy. Future research in teaching Einsteinian energy at this level should further explore the results of our study, providing more information on students’ conceptions and teaching practices.

## Data Availability

The data that support the findings of this study are available from the corresponding author, [S.B.], upon reasonable request.
